# Host–guest interaction and properties of cucurbit[8]uril with chloramphenicol

**DOI:** 10.3762/bjoc.17.194

**Published:** 2021-12-03

**Authors:** Lin Zhang, Jun Zheng, Guangyan Luo, Xiaoyue Li, Yunqian Zhang, Zhu Tao, Qianjun Zhang

**Affiliations:** 1 Key Laboratory of Macrocyclic and Supramolecular Chemistry of Guizhou Province, Guizhou University, Guiyang 550025, China

**Keywords:** antibacterial activity, chloramphenicol, cucurbit[8]uril, host–guest interaction, in vitro cumulative release, stability

## Abstract

The interaction between cucurbit[8]uril (Q[8]) and chloramphenicol (CPE) was investigated using single-crystal X-ray diffraction spectroscopy, isothermal titration calorimetry (ITC) and UV–vis, NMR and IR spectroscopy. The effects of Q[8] on the stability, in vitro release performance and antibacterial activity of CPE were also studied. The results showed that CPE and Q[8] formed a 1:1 inclusion complex (CPE@Q[8]) with an inclusion constant of 5.474 × 10^5^ L/mol. The intervention of Q[8] did not affect the stability of CPE, but obviously reduced the release rate of CPE in artificial gastric and intestinal juice; Q[8] has a slow-release effect on CPE. The antibacterial results showed that the minimum inhibitory concentration (MIC) of CPE and CPE@Q[8] toward *Escherichia coli* (*E. coli*) was 1.5 × 10^–3^ and 1.0 × 10^–3^ mol/L, respectively, and toward *Staphylococcus aureus* (*S. aureus*), the MIC was 2.0 × 10^–3^ mol/L for both CPE and CPE@Q[8]. Therefore, Q[8] enhanced the inhibitory activity of CPE against *E. coli*.

## Introduction

**Figure 1:**
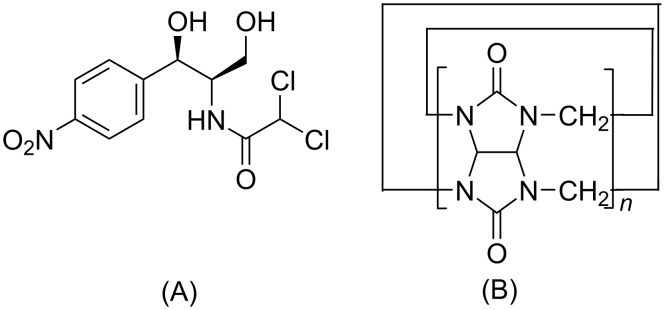
The structures of chloramphenicol (A) and cucurbit[*n*]urils (B).

Chloramphenicol (CPE, Figure 1A) is a broad-spectrum antibiotic resulting from the metabolism of chorismic acid in *Streptomyces venezuelae* [1], which has a certain inhibitory effect on many Gram-positive and -negative cocci bacteria, as well as anaerobic bacteria [2], and is used for the treatment of typhoid, meningitis, chlamydia, eye infections, purulent wounds and other diseases [3]. Chloramphenicol is slightly soluble in water and has a bitter taste. Upon forming an inclusion complex with cyclodextrin, the solubility and bitter taste of CPE can be improved [4–5]. Ramesh Gannimani et al. [6] reported that the inclusion complex of cyclodextrin and CPE loaded silver nanoparticles possessed stronger antibacterial properties than CPE alone. Ana I. Ramos et al. [7] studied the inclusion compound of CPE and cyclodextrin and reported its effect on Enterococcus, Bacillus, Staphylococcus and other bacteria.

As a new type of supramolecular host compound, cucurbit[*n*]urils [8–19] (Q[*n*]s, Figure 1B) form via the polymerization of multiple glycoside units. The ports on both sides of these compounds are surrounded by carbonyl oxygen atoms, which form a hydrophobic cavity and two hydrophilic ports [20]. The outer surface of the cucurbit[*n*]uril is composed of a large number of nitrogen atoms and carbon atoms and the cavity has a certain degree of hydrophobicity that can form a stable host–guest inclusion complex with a guest molecule via non-bonding interactions, such as hydrogen bonds, van der Waals forces and ionic dipoles [21–28]. It has been proved that cucurbit[*n*]urils can be used as non-toxic and safe drug carriers [29–31], among which cucurbit[8]uril (Q[8]) [32] has a large cavity. Q[8] interacts with a variety of small drug molecules such as chrysin, oroxin A and B, baicalein, etc., which can enhance the solubility, stability, antioxidant and sustained-release ability of drug molecules [31,33–34]. However, previous studies rarely reported the interaction between Q[8] and antibiotics, and did not explore the effect of Q[8] on antibacterial activity of antibiotics. Herein, Q[8] was selected as the host and the host–guest interaction between Q[8] and CPE was studied using single-crystal X-ray diffraction, UV–vis and ^1^H NMR spectroscopy, and the effects of Q[8] on the stability, in vitro release rate and antibacterial activity of CPE were investigated.

## Results and Discussion

### The host–guest interaction between Q[8] and CPE

#### Single-crystal structure analysis of CPE@Q[8]

**Figure 2:**
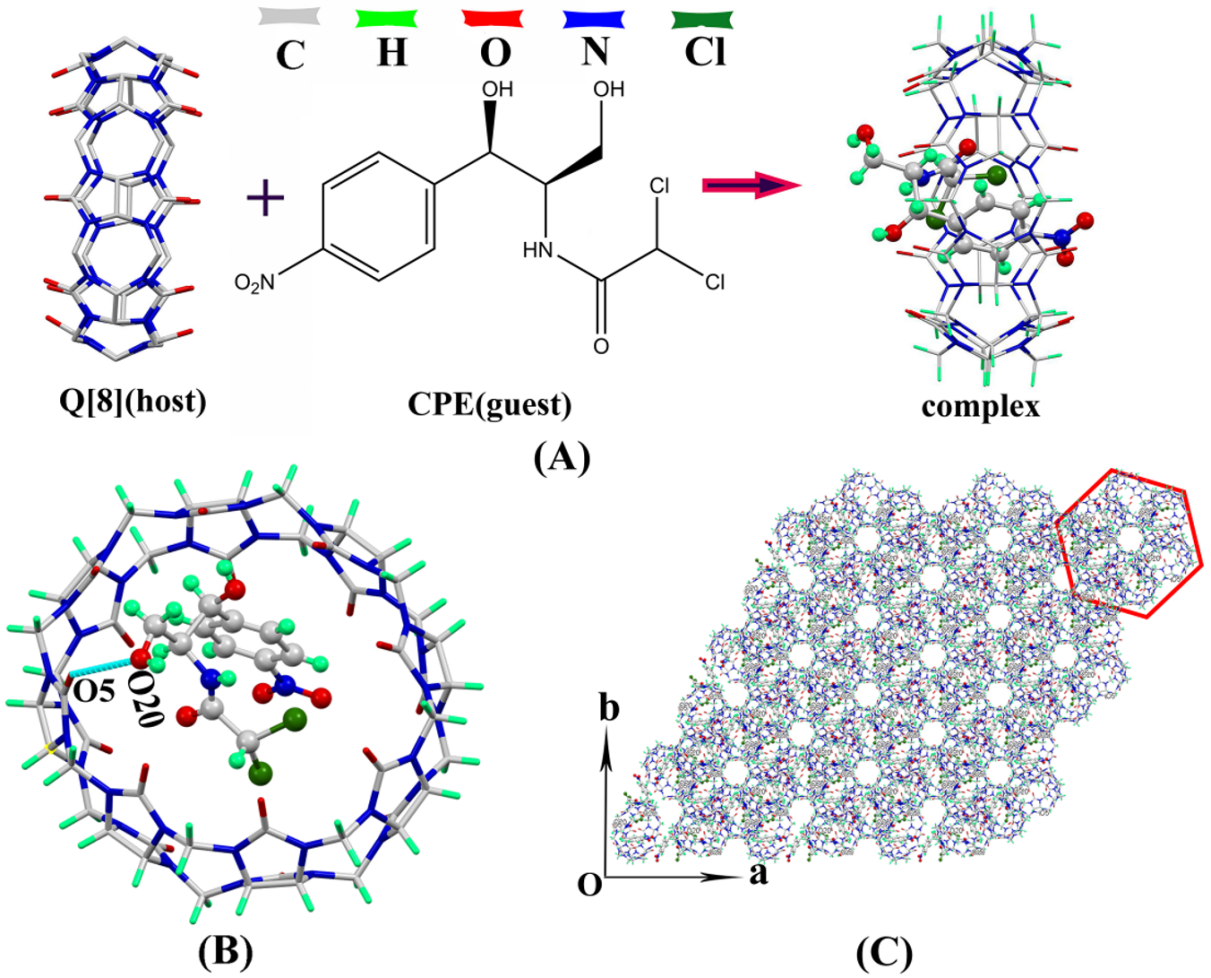
(A) CPE and Q[8] structural model diagram, (B) interaction between CPE and Q[8], (C) CPE@Q[8] stacked graph along the c-axis.

The clathrate mode and crystal parameters of CPE and Q[8] were determined on a Bruker D8 Venture single-crystal diffractometer and shown in Figure 2 and Supporting Information File 1, Table S1, respectively. Figure 2A shows the interaction of CPE and Q[8] results in an asymmetric CPE@Q[8] structure, including one Q[8] and one CPE molecule and the entire CPE molecule enters the Q[8] cavity. Therefore, Q[8] and CPE form a 1:1 host–guest inclusion complex (CPE@Q[8]). Figure 2B shows the O20 atom of the CPE molecule and the port oxygen atom (O5) of Q[8] interact through the formation of a O–H···O hydrogen bond, in which the bond distance between O20 and O5 was 2.921 Å. It can be seen that the CPE molecule is distorted at the C51 atom and the bond angle between C53–C51–N34 was 103.30°, which makes the CPE molecule fixed and enter the cavity of Q[8]. Figure 2C shows the crystal structure stacking diagram of the CPE@Q[8] host–guest complex along the *c*-axis. It can be clearly seen that small hexagonal holes are formed between the complexes, which are expected to have potential applications in molecular adsorption and drug delivery.

#### UV–visible spectroscopy

**Figure 3:**
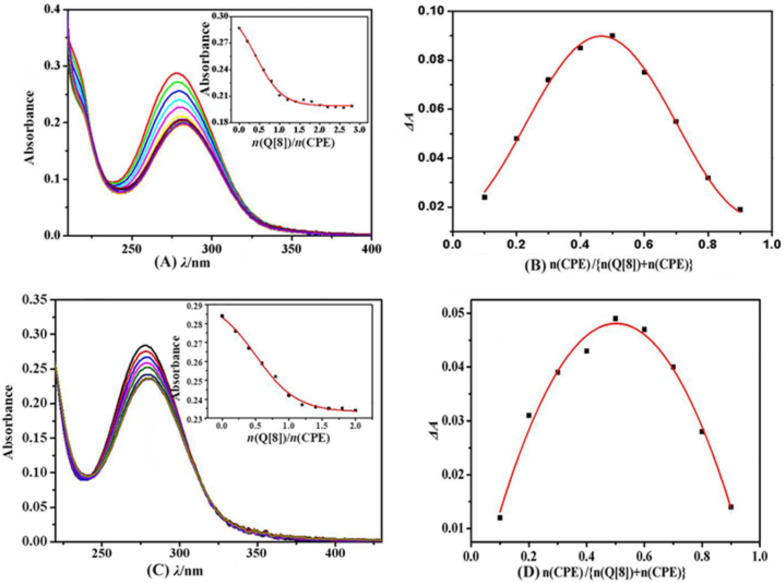
UV–vis absorption spectra of CPE with Q[8] in aqueous solution (A) or hydrochloric acid solution (C) and UV–vis Job’s plot of ΔA against *n*(Q[8])/[*n*(Q[8]) + *n*(CPE)] at 278 nm (B, D). *c*_(CPE)_ = 30 μmol/L, (*c*_(Q[8])_/*c*_(CPE)_) = 0, 0.2, 0.4, 0.6, 0.8, 1.0; ··· 2.8), insets in (A, C) are the plot of absorbances at 278 nm of CPE.

The interaction between Q[8] and CPE was investigated using UV–vis spectroscopy utilizing the molar ratio and Job's method under neutral conditions (Figure 3). Figure 3A shows that CPE has a strong absorption peak at λ = 278 nm and the absorption intensity gradually decreases after the continuous addition of Q[8]. When *n*(Q[8])/*n*(CPE) = 1:1, the absorbance exhibits an obvious turning point and further increasing the concentration of Q[8] does not change the absorption of the system. The spectrogram determined using Job's method is shown in Figure 3B. When *n*(Q[8])/[*n*(Q[8]) + *n*(CPE)] = 0.5, the maximum value of ΔA appears, indicating the formation of a 1:1 host–guest inclusion complex.

Due to the poor solubility of Q[8], an acid solution was selected as the medium to grow a crystal of the CPE@Q[8] host–guest inclusion complex. When the interaction between CPE and Q[8] was investigated using ^1^H NMR spectroscopy, a deuterated hydrochloric acid solution (V_D2O_/V_DCl_ = 3:2) was used as the NMR solvent. Consequently, the interaction between Q[8] and CPE in hydrochloric acid solution was studied (Figure 3C and D). The results show that the molar ratio of CPE and Q[8] was 1:1 under acidic conditions, which was the same as that observed under neutral aqueous conditions.

#### ITC study of the interaction between CPE and Q[8]

**Figure 4:**
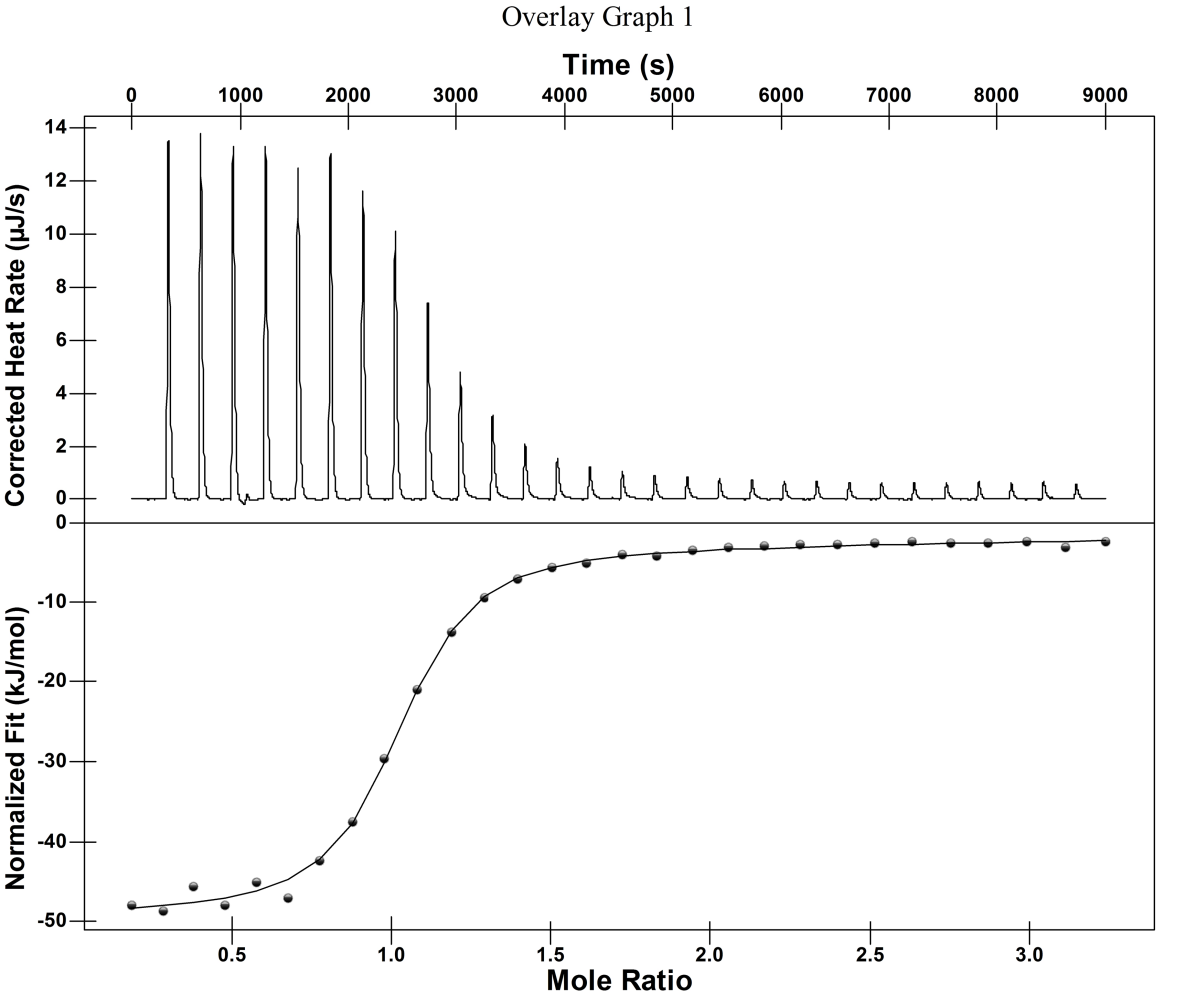
ITC data obtained for the binding of Q[8] with CPE in an aqueous solution at 25 °C.

ITC is a highly sensitive and automated microcalorimeter method, which can continuously and accurately monitor and record the calorimetric curve of each process to obtain the thermodynamic parameters and action ratio between the assemblies. Figure 4 and Table 1 show the exothermic isotherms and thermodynamic constants obtained for the titration of CPE with Q[8] interaction using ITC. From the data, it can be seen that the reaction was enthalpy driven and its binding constant was 8.057 × 10^5^ L/mol.

**Table 1:** Thermodynamic parameters related to the CPE@Q[8] system at 25 °C.

Complex	K [L·mol^−1^]	*∆G* [kJ·mol^−1^]	*∆H* [kJ·mol^−1^]	*T∆S* [kJ·mol^−1^]

CPE@Q[8]	8.057 × 10^5^	−34.63	−43.53	11.66

#### ^1^H NMR spectroscopy

**Figure 5:**
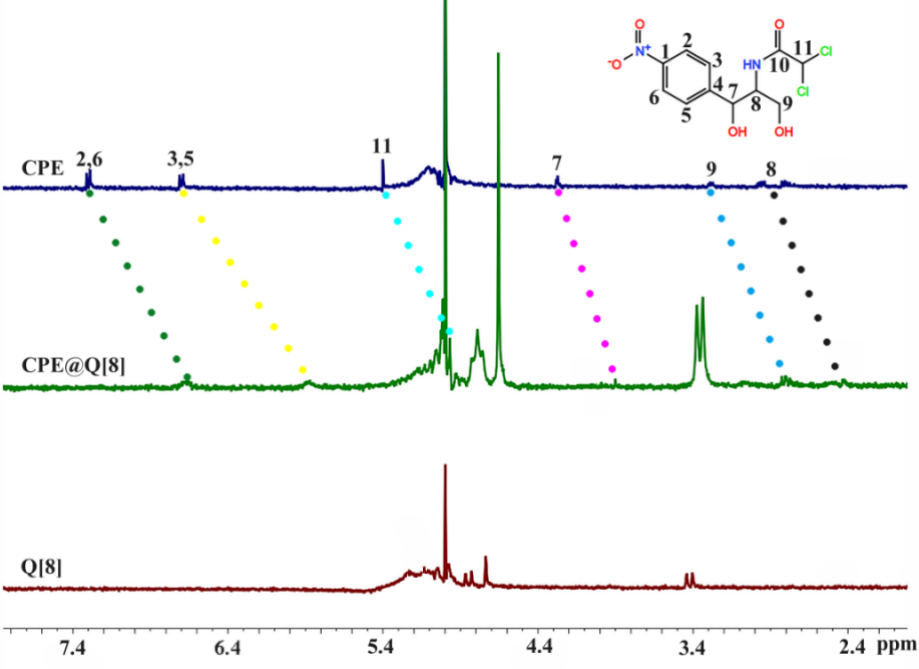
^1^H NMR spectra of CPE, CPE@Q[8] and Q[8] (V_D2O_/V_DCl_ = 3:2).

^1^H NMR spectroscopy is one of the most important methods used to explore the host–guest interaction mode. Through the interaction of the cucurbit[*n*]uril on the guest molecule, the chemical shift of the proton resonance peaks of the guest can be observed in water, so a mixed solution of deuterated hydrochloric acid and deuterated water was used as the NMR solvent. After adding Q[8], the chemical shifts of all proton resonance peaks of CPE are shifted toward the high field region, as shown in Figure 5 and Table 2. So it can be inferred that the whole CPE molecule enters the cavity of Q[8], which is consistent with our single-crystal X-ray analysis of CPE@Q[8] shown in Figure 2.

**Table 2:** Changes in^1^H NMR chemical shift of CPE after the addition of Q[8] (V_D2O_/V_DCl_ = 3:2).

^1^H nucleus	2-H, 6-H	3-H, 5-H	7-H	8-H	9-H	11-H

Δδ/ppm^a^	−0.64	−0.81	−0.39	−0.24	−0.23	−0.57

^a^The formula for calculating the chemical shift of the proton resonance peak caused by the coordinated combination of CPE and Q[8] is as follows: Δδ = δ_complex −_ δ_free._

#### IR spectroscopy

**Figure 6:**
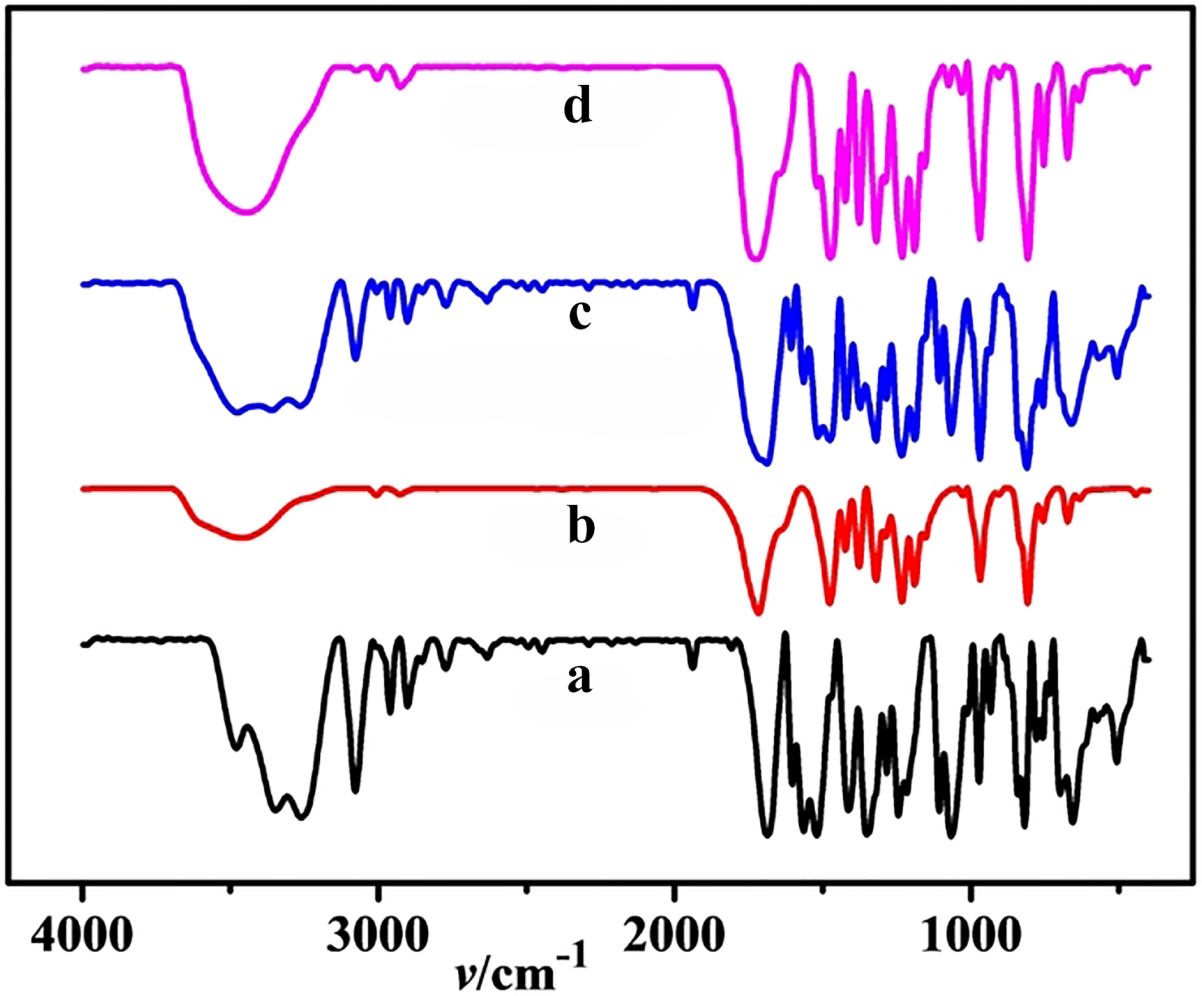
IR spectra recorded for Q[8] (a), CPE (b), a physical mixture of Q[8] and CPE (c), and the CPE@Q[8] inclusion complex (d).

Figure 6 shows the IR spectra recorded for Q[8] (a), CPE (b), a physical mixture of Q[8] and CPE {*n*(Q[8])/*n*(CPE) = 1:1} (c) and the CPE@Q[8] inclusion complex (d). By comparison, spectrum (c) is a simple superposition of the spectra recorded for Q[8] (a) and CPE (b), and there was no interaction. When comparing (c) and (d), the C–H stretching vibration peak was observed at 3100 cm^−1^ and the C=C skeleton vibration peaks of the benzene ring of CPE were observed at 1603, 1520 and 1413 cm^−1^; the bending vibration peaks of the O–H bonds were observed at 1106 and 1066 cm^−1^. The nitro-symmetric tensile vibration peak observed at 1503 cm^−1^ and the nitro-asymmetric tensile vibration peak at 1320 cm^−1^ disappeared in the spectrum (d). At the same time, the fingerprint region peak of the benzene ring observed from 500 to 900 cm^−1^ disappeared or weakened. Therefore, it can be inferred that CPE interacts with Q[8].

### The effect of Q[8] on the properties of CPE

#### Stability analysis

**Figure 7:**
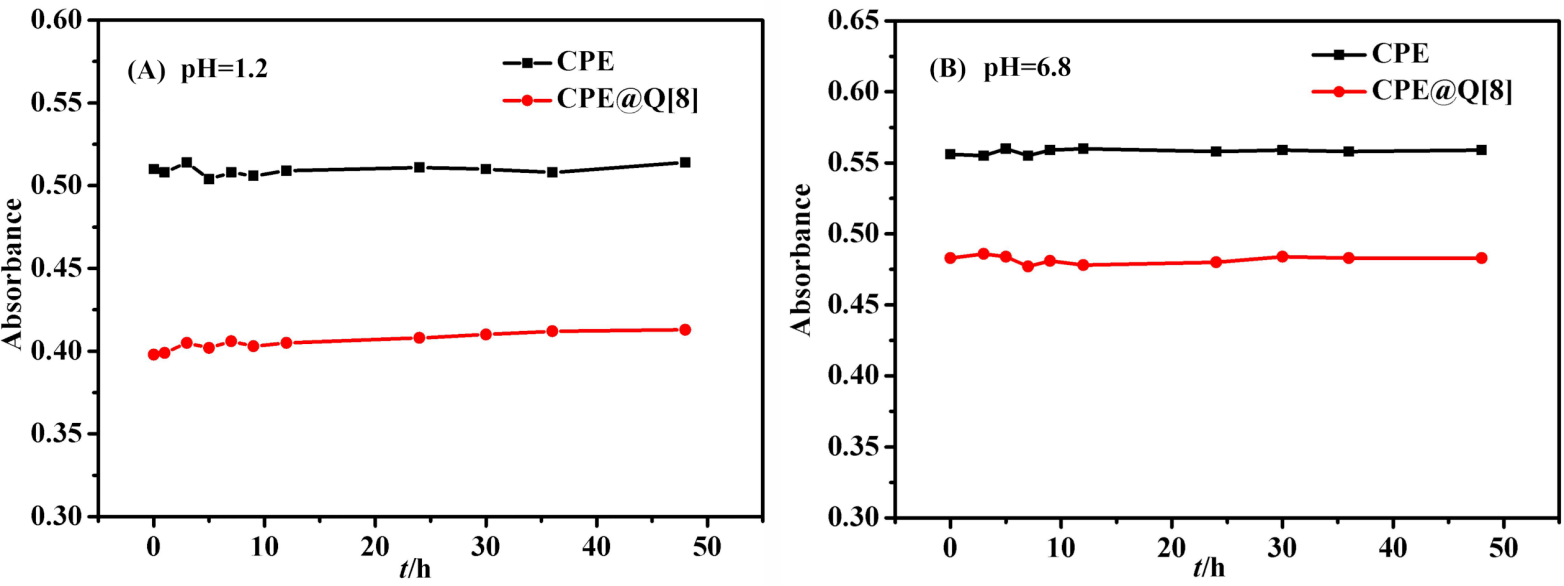
UV absorption intensity of CPE and CPE@Q[8] changes with time in the artificial gastrointestinal juice (pH 1.2, pH 6.8).

The stability of CPE and CPE@Q[8] in artificial gastrointestinal juice was investigated using UV–is spectroscopy. Figure 7A shows the variation of the UV absorption intensity of CPE and CPE@Q[8] over time in simulated gastric juice (pH 1.2). Figure 7B shows the relationship between the UV absorption intensity of CPE and its inclusion complex in artificial intestinal fluid (pH 6.8) with time. The results show that CPE itself has high stability in artificial gastrointestinal juice and the intervention of Q[8] did not change its stability.

#### Drug release behavior in vitro

**Figure 8:**
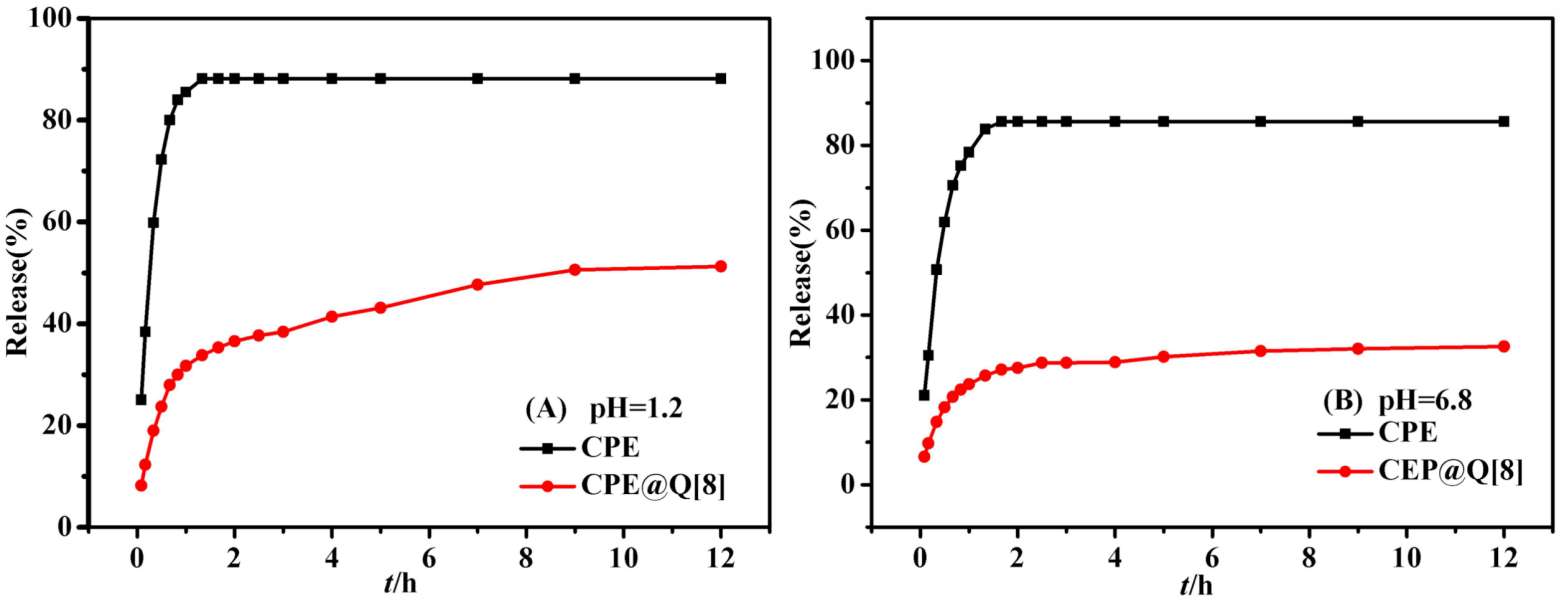
Release curve of CPE and CPE@Q[8] in artificial gastrointestinal juice (pH 1.2, pH 6.8).

Figure 8 shows the release curve obtained for CPE and its inclusion complex in artificial gastrointestinal juice. Figure 8A shows that CPE was completely released after 1.3 h in artificial gastric juice (pH 1.2) and its release rate was 88.19%. CPE@Q[8] basically reached a release equilibrium after 9 h and the cumulative release rate was 51.26%. It is possible that the inclusion of CPE in Q[8] causes its release rate to be reduced and has a slow-release effect. In artificial intestinal fluid, CPE reached the release end point after 1.67 h, and its in vitro cumulative release rate was 85.63% (Figure 8B). The release rate of CPE@Q[8] was faster before 2 h, but the release rate was slow after 2 h, and its cumulative release rate was 32.59% after 12 h. The results show that the incorporation of CPE in Q[8] has a slow-release effect on the artificial gastrointestinal juice.

#### Antibacterial activity

The minimum inhibitory concentration (MIC) of CPE and CPE@Q[8] was determined using the test tube double dilution method (Table 3). The results showed that the MIC of CPE and CPE@Q[8] against *E. coli* was 1.5 × 10^−3^ and 1.0 × 10^−3^ mol/L, respectively. The intervention of Q[8] increased the anti-*E. coli* effect of CPE by 1.5 times. The MIC values of CPE and CPE@Q[8] on *S. aureus* were both 2.0 × 10^−3^ mol/L, and the intervention of Q[8] had no effect on CPE against *S. aureus*.

**Table 3:** The minimum effective concentration (MIC) of CPE, CPE@Q[8] against *E. coli* and *S. aureus*.

	MIC [mol/L ]	MIC [mol/L ]
Sample	*E. coli*	*S. aureus*

CPE	1.5 × 10^−3^	2.0 × 10^−3^
CPE@Q[8]	1.0 × 10^−3^	2.0 × 10^−3^

## Conclusion

Herein, the 1:1 host–guest complex of CPE and Q[8] was confirmed using single-crystal X-ray diffraction and ^1^H NMR, UV–vis and IR spectroscopy. The CPE molecule completely enters the cavity of Q[8] with an inclusion constant of 5.474 × 10^5^ L/mol. The intervention of Q[8] has no effect on the stability of CPE, which has a slow-release effect on CPE in artificial gastrointestinal juice and improves the inhibitory ability of CPE against *E. coli*. Our experimental results provide a theoretical basis for the application of CPE.

## Supporting Information

File 1 Experimental part.
