# Supplementary material for: Host–guest interaction and properties of cucurbit[8]uril with chloramphenicol
Source: Beilstein J Org Chem. 2021 Dec 3;17:2832–9. doi: 10.3762/bjoc.17.194 (PMC8649205; doi:10.3762/bjoc.17.194)
Supplement: File 1 — Experimental part. [file Beilstein_J_Org_Chem-17-2832-s001.pdf]

BEILSTEIN JOURNAL OF ORGANIC CHEMISTRY

Supporting Information

for

Host–guest interaction and properties of cucurbit[8]uril with chloramphenicol

Lin Zhang, Jun Zheng, Guangyan Luo, Xiaoyue Li, Yunqian Zhang, Zhu Tao and Qianjun Zhang

Beilstein J. Org. Chem. **2021,** *17,* 2832–2839. doi:10.3762/bjoc.17.194

Experimental part

License and Terms: This is a supporting information file under the terms of the Creative Commons Attribution License (https://creativecommons.org/licenses/by/4.0). Please note that the reuse, redistribution and reproduction in particular requires that the author(s) and source are credited and that individual graphics may be subject to special legal provisions.
The license is subject to the *Beilstein Journal of Organic Chemistry* terms and conditions: (https://www.beilstein-journals.org/bjoc/terms)

Table of contents

1 Reagents....S2
2 Apparatus S2
3 Methods....S3
3.1 CPE@Q[8] crystal preparation and determination....S3
3.2 UV-Visible Spectroscopy Analysis S4
3.3 Isothermal titration calorimetry (ITC) measurements....S4
3.4 ^{1}H NMR spectroscopy S4
3.5 IR spectra analysis S5
3.6 Stability analysis S5
3.7 In vitro release studies....S5
3.8 Antibacterial activity S5
4 references S6

1 Reagents

Q[8](purity≥97%) was prepared in the Key Laboratory of Macrocyclic and Supramolecular Chemistry of Guizhou Pro- vince, China. CPE (purity≥97%) was purchased from Shanghai Yuanye Biological Co., Ltd). U.S. imported dialysis bags (Lot number: MD44; molecular weight cut-off: 500 Da) were purchased from Shanghai Fangxiao Biological Technology Co., Ltd. Deionized water was used throughout our studies. Nutrient agar (33 grams of configured medium per 1000 milliliters of water) was purchased from Shanghai Bo Microbiology Technology Co., Ltd. Escherichia coli (The laboratory of the School of Pharmacy of Guizhou University is subcultured by itself). Staphylococcus aureus (The laboratory of the School of Pharmacy of Guizhou University is subcultured by itself). The preparation of CPE@Q[8] inclusion compound: According to the ratio of n(Q[8]):n(CPE) =1:1, weigh 0.02 g of Q[8] and 0.1145 g of CPE to solutions A and B with deionized water as the solvent (A:$c_{(CPE)}$ =1.0×10^{-3} mol/L, B:$c_{(Q[8])}$=1.0×10^{-4} mol/L). And then mix and stir for 1 h. The solvent was removed in vacuo to obtain the CPE@Q[8] inclusion compound .

2 Apparatus

UV-2700 dual-beam ultraviolet-visible (UV–vis) spectrophotometer (Shimadzu Instruments Co., Ltd., it was used to measure the absorbance of the substance under test to visible light or ultraviolet light (200–760nm) and perform quantitative analysis.); PHS-25 digital pH meter (Shanghai INESA Scientific Instrument Co., Ltd., it was mainly used to accurately measure the pH value of liquid media.); AKHL-III-08 Eco laboratory ultrapure water machine (Chengdu Eco Water Treatment Equipment Co., Ltd.; an instrument for preparing deionized water); FA2204N Electronic Balance (Shanghai Jinghai Instrument Co., LTD., Instrument for weighing sample mass); Nano ITC isothermal calorimeter (TA company, USA, An instrument that measures the interaction between biomolecules, such as binding constant KB,

measurement number n, entropy S, enthalpy H and other parameters); JNM-ECZ400s MHz Nuclear Magnetic Resonance System (JEOL); SHA-IIIS constant temperature oscillator (Zhengzhou Great Wall Technology Industry and Trade Co., Ltd.); 101-1AB electric heating blast drying oven (Tianjin Test Instrument Co., Ltd.); Bruker D8 VENTURE diffractometer; high temperature and pressure Sterilization pot (Shanghai Sanshen Instrument Co., Ltd.); DH3600 electric heating constant temperature incubator（Tianjin Test Instrument Co., Ltd.）

3 Methods

3.1 CPE@Q[8] crystal preparation and determination

Weigh 0.01 g Q[8] and 0.01 g CPE, add 1.5 mL (0.1 mol/L）formic acid aqueous solution to dissolve, and obtain transparent crystals after standing for a period of time. Then the crystals were collected and tested by a Bruker D8 VENTURE diffractometer, and the crystals were analyzed. The crystal parameters and data collection conditions of CPE@Q[8] are measured (Table S1). All parameters of this crystal have been saved in the Cambridge Crystallography Data Center as a supplementary publication number CCDC: 2071782.

Table S1 X-ray crystal data obtained for CPE@Q[8]

Empirical formula	$C_{59}H_{62}O_{22}Cl_2N_{34}$	Z	9
M_r	1670.32	Dc (g cm $^{-3}$)	1.295
Crystal system	trigonal	F(000)	7776
Space group	R 3	μ (mm $^{-1}$)	0.161
a (Å)	29.714(7)	Params	1055
b (Å)	29.714(7)	R_{int}	0.1186
c (Å)	25.202(11)	$R[I > 2\sigma(I)]$[a]	0.0805
α (deg)	90	$wR[I > 2\sigma(I)]$[b]	0.2199
β (deg)	90	R(all data)	0.1134
γ (deg)	120	wR(all data)	0.2434
V [Å^3]	19270(12)	GOF (F^2)	1.036

[a] Conventional R on Fhkl: $\sum||F_o| - |F_c||/\sum|F_o|$.

[b] Weighted R on $|Fhkl|^2$: $\sum[w(F_o^2 - F_c^2)^2]/\sum[w(F_o^2)^2]^{1/2}$.

3.2 UV–visible spectroscopy analysis

Deionized water (or hydrochloric acid solution, pH=1.0) was used as the solvent, and CPE with a concentration of 1.0 × 10^{-3} mol/L and Q[8] with a concentration of 1.0 × 10^{-4} mol/L were prepared for reserve. Mole ratio method and Job's method were used to investigate the interaction mole ratio of CPE and Q[8], respectively. The molar ratio method is to add 300 μL of CPE aqueous solution to 10 mL volumetric flasks, and the molar ratio of *n*(Q[8])/*n*(CPE) is 0, 0.2, 0.4, 0.6, 0.8, 1.0, 1.2, 1.4 , 1.6, 1.8, 2.0, 2.2, 2.4, 2.6, 2.8, respectively add the corresponding amount of Q[8] and fix the volume. After 1 h placement, the UV absorption intensity was measured. Job's method is based on n(Q[8])/{n(Q[8])+n(CPE)}= 0, 0.1 ··· 0.8, 0.9, deionized water is used as the solvent, placed for 1 h after configuration, and measured UV absorption intensity.

3.3 Isothermal titration calorimetry (ITC) measurements

Using a method similar to that reported by Jin Y. M.[1] et al., thermodynamic parameters and binding constants (*K*) were determined by isothermal titration calorimeter Nano ITC (TA, USA). An 1.0 × 10^{-3} mol·L^{-1} CPE and 1.0 × 10^{-4} mol·L^{-1} Q[8] solution were prepared using deionized water. Q[8] was titrated with the CPE solution at 25 °C, 300 s, 8 μL·d^{-1}, stirring speed of 250 r·min^{-1}, and the thermodynamic coefficient of the system was determined.

3.4 ^{1}H NMR spectroscopy

Using the mixed solution (V_{D2O}/V_{DCl} = 3:2) as the solvent, and to study the ^{1}H NMR spectrum of the interaction between CPE and Q[8]. ^{1}H NMR spectra were recorded at 20 °C on a JNM-ECZ400s MHz nuclear magnetic resonance (NMR) spectrometer.

3.5 IR spectra analysis

CPE, Q[8], a physical mixture of CPE and Q[8] ($n_{(Q[8])}/n_{CPE}$ = 1:1) and CPE@Q[8] were weighed, respectively. KBr was added to prepare KBr discs of the samples to record the IR spectra over a wavenumber range of 4000–500 cm^{-1}.

3.6 Stability analysis

The UV absorption intensity curves of CPE and CPE@Q[8] with the concentration of 3.0×10^{-5} mol/L were measured in the artificial gastric juice (pH = 1.2) or artificial intestinal juice (pH = 6.8) system, respectively.

3.7 In vitro release studies

The isothermal oscillation method[2] was used to study the in vitro release behavior of the inclusion compound. After accurately weighing 0.009 mmol of CPE and 0.009 mmol of CPE@Q[8]. The samples were added to dialysis bags and placed in a thermostatic shaker containing artificial intestinal fluid (pH = 6.8 phosphate buffer solution) or artificial gastric juice (pH = 1.2 hydrochloric acid solution), shaken slowly in a water bath at 37 °C. At appropriate time intervals, 3 mL of each sample was removed, adding the same volume of fresh release medium at the same time. According to the working curve to calculate the amount of drug released, the absorbance of the samples was measured. The degree of release is calculated by the following formula:

Release degree (%) = (Actual release of CPE / The total mass of CPE) ×100%

3.8 Antibacterial activity

The determination method adopts the test tube double dilution method[3], Each sample uses 8 test tubes, numbered in sequence, of which the first one is used as a blank control (no bacteria inoculated) , the second branch is used as a solvent control (containing bacterial culture solution), and the remaining 6 tubes are diluted two-fold

with liquid culture medium. Add 30 μL of bacterial suspension to each tube, culture in a shaker (200 r/min) at 28 °C for 48 h, then take out and compare with the blank control tube for observation. The drug concentration corresponding to no turbidity is the sample vs. the tested bacteria Minimum inhibitory concentration MIC).

4 References

[1] Jin, Y. M.; Jiang D. F.; Meng Y.; Gao J.; Zheng J.; Ma P. H.; *J. Incl. Phenom Macro.* **2021,** 100, 209-215.

[2] Xu, Z. L.; Lian, X. W.; Li, M. J. *Chem. Res. Chinese U.* **2017,** 33, 1-6.

[3] Yu, S. R. *second edition. Beijing: People's Medical Publishing House*, **200**2, 452-458.
